# Estimating healthy life years without activity limitations using medical claims data in Japan

**DOI:** 10.1093/ije/dyag126

**Published:** 2026-07-30

**Authors:** Masahiro Nishi, Reo Nagamitsu, Satomi Morita, Satoaki Matoba

**Affiliations:** Department of Cardiovascular Medicine, Graduate School of Medical Science, Kyoto Prefectural University of Medicine, Kyoto, Japan; Department of Health and Welfare, Kyoto Prefectural Government, Kyoto, Japan; Department of Epidemiology for Community Health and Medicine, Graduate School of Medical Science, Kyoto Prefectural University of Medicine, Kyoto, Japan; Department of Health and Welfare, Kyoto Prefectural Government, Kyoto, Japan; Department of Pulmonary Medicine, Graduate School of Medical Science, Kyoto Prefectural University of Medicine, Kyoto, Japan; Department of Cardiovascular Medicine, Graduate School of Medical Science, Kyoto Prefectural University of Medicine, Kyoto, Japan

**Keywords:** healthy life years, healthy life expectancy, medical claims data, National Health Insurance, machine learning, health disparities

## Abstract

**Background:**

Healthy life years are estimated from national population surveys that assess limitations in daily activities. However, the infrequent timing and limited sample sizes hinder the timely and detailed assessment of regional health conditions. We aimed to develop a novel method to estimate municipal-level healthy life years without activity limitations by using medical claims data.

**Methods:**

We analysed medical claims data in Kyoto Prefecture, Japan. Outpatient data from May to July in 2016 and 2019 (*n* = 1 489 920) were used for development and data from the same period in 2022 (*n* = 739 236) were used for evaluation. A total of 5743 diagnostic codes were aggregated into 40 disease categories. A machine-learning model was employed to produce the probability of activity limitation, which was calibrated by using data from the Comprehensive Survey of Living Conditions and applied to derive age-specific prevalence rates. Healthy life years at the municipal level as of June 2022 were then estimated by incorporating a life table.

**Results:**

We observed variation in healthy life years among municipalities: 72.1 years across all regions for males, ranging from 67.3 to 75.2 years, and 75.8 years for females, ranging from 71.3 to 77.6 years. Regional disparities were also noted in the prevalence of diseases associated with activity limitations.

**Conclusion:**

This study provides the first robust, scalable method to estimate healthy life years without activity limitations at the municipal level by using real-world administrative data. Timely monitoring of regional healthy life years will support targeted health-promotion policies and contribute to reducing health disparities.

Key Messages

- We aimed to establish a method for estimating healthy life years without activity limitations in real time at the municipal level by using administrative claims data.
- We found substantial disparities across municipalities that traditional national surveys were unable to detect.
- Our approach offers a scalable tool for regional health monitoring, supporting the timely implementation of health policies and evidence-based interventions to reduce geographic health inequalities.

## Introduction

In aging societies, there is growing interest in healthy life years, also referred to as healthy life expectancy, which is defined as the period during which individuals can live without limitations in daily activities due to health-related issues. It is well established that lifestyle-related diseases and mental health disorders negatively impact healthy life years [1–3]. Recent studies utilizing machine-learning techniques have identified several key determinants of healthy life years, including aging, mental disorders such as depression, musculoskeletal conditions, neurological diseases, and other chronic illnesses [4]. To promote health and further extend healthy life years, it is essential to enhance physical activity through daily walking and regular exercise, maintain a balanced diet, and support mental conditions [5–7].

Regional disparities in life expectancy and healthy life years have been documented across various geographic units, including countries, prefectures, and municipalities [8–11]. Beyond lifestyle and disease-related factors, socioeconomic status also plays a significant role in shaping geographic health inequalities [10, 11]. Reducing these regional disparities requires the timely implementation of effective health policies that address the complex, region-specific contributing factors.

In Japan and Western countries, national surveys are conducted to estimate healthy life years based on limitations in daily activities or self-rated health status [12–14]. However, the several-year interval between the surveys makes it impossible to promptly evaluate changes in healthy life years during this period. Additionally, there is a time lag of years before the survey results are published. These delays hinder the real-time assessment of regional health challenges. Healthy life years are calculated at the national, prefectural, and major-city levels, but not at the municipal level due to the limited sample size of the survey. Furthermore, there has been no established method to evaluate healthy life years without activity limitations based on medical claims data, which serve as real-world electronic administrative records. In this study, we aimed to develop a novel method to evaluate monthly regional healthy life years without activity limitations by using medical claims records.

## Methods

### Data description

We utilized the medical claims data of all members participating in the National Health Insurance (commonly known as KOKUHO) and the Medical Care System for Elderly in the Latter Stage of Life in Kyoto Prefecture, Japan, in 2016, 2019, and 2022. This system is cooperatively operated by prefectural and municipal governments. The database comprises ∼38.5% of all residents in Kyoto Prefecture, including people aged ≥75 years. The municipality represents the area in which the insured persons reside. From the database, we used outpatient data from the 3 months from May through to July in 2016 and 2019 (*n* = 1 489 920) for the development of the estimation method and the data from 2022 (*n* = 739 236) for the evaluation.

In addition to the medical claims data, we incorporated information from the Comprehensive Survey of Living Conditions (CSLC)—a nationwide cross-sectional survey conducted every 3 years by the Ministry of Health, Labour and Welfare of Japan. This survey aims to assess a broad range of topics related to the population’s well-being, including healthcare, welfare services, pensions, and income status [12]. For this study, we utilized health-related responses from 17 215 participants residing in Kyoto Prefecture, collected in June 2016 and 2019, to calibrate our model estimates. Data used in the analysis included respondents’ activity limitations, age, sex, and the presence of any of 40 disease or injury categories for which they were receiving treatment.

### Model description

We employed the machine-learning model that we had previously developed to generate the probability of individual activity limitation [4]. We selected the extreme gradient boosting (XGB) classifier with hyperparameters (n_estimators = 200, max_depth = 9, eta = 0.1, min_child_weight = 2, max_delta_step = 5, and subsample = 0.5) that yielded the highest area under the receiver-operating characteristic curve. Furthermore, the predictors included age, sex, and 40 disease categories.

Medical claims data were extracted by using SQL Server 2020. A machine-learning model was implemented with Python version 3.12.2 to estimate the individual probabilities of experiencing activity limitations, based on a previously reported method [4]. Statistical analyses were performed by using R version 4.2.0 [15].

### Algorithmic flow of the estimation method for regional healthy life years without activity limitations by using medical claims data

**Figure 1:**
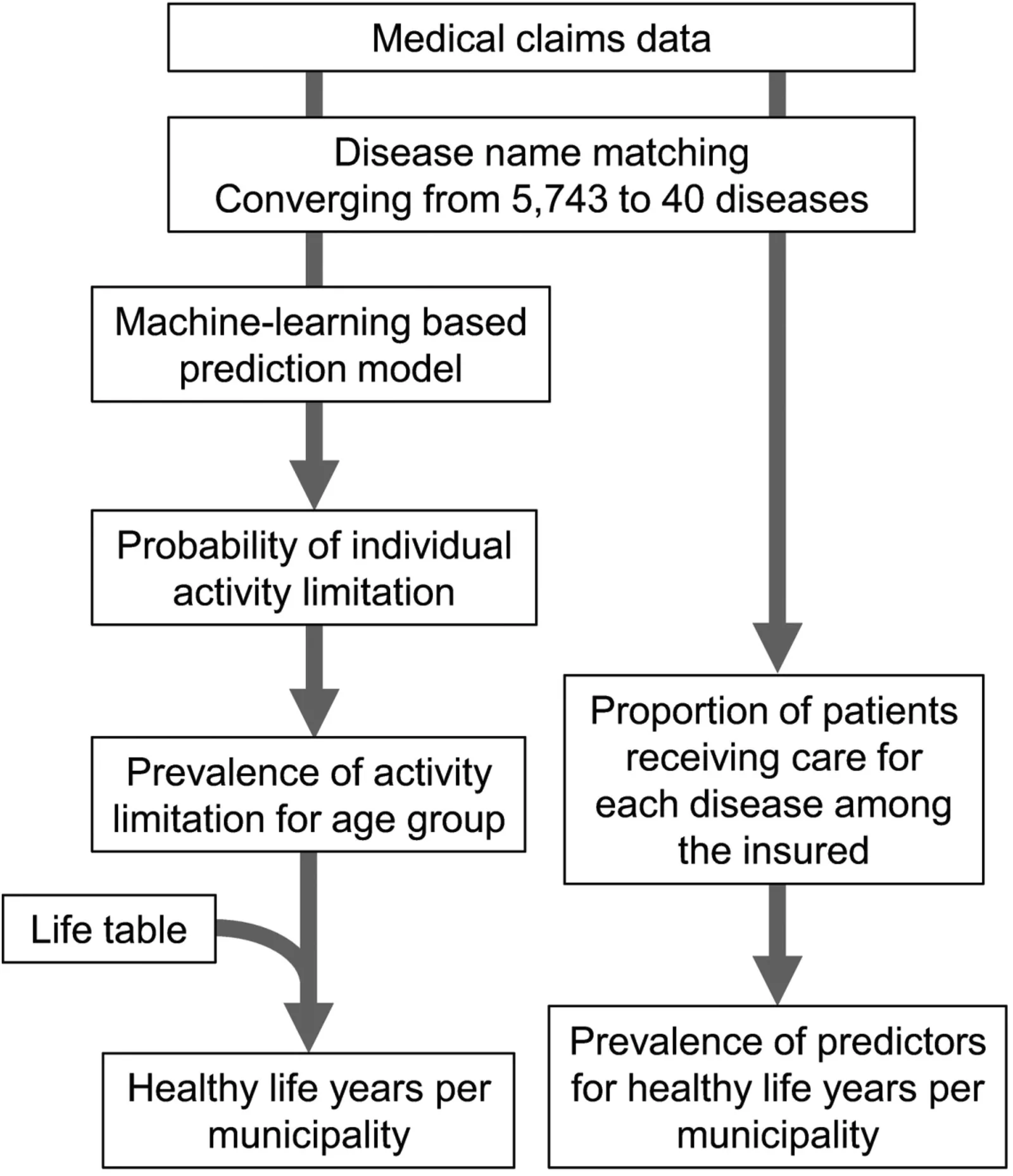
Flow diagram of the estimation method for healthy life years without activity limitations by using medical claims data. The flow is divided into two arms. The left arm of the algorithm represents the process for estimating healthy life years, whereas the right arm represents the process for estimating the prevalence of diseases affecting healthy life years.

The algorithmic flow of the estimation method for healthy life years without activity limitations by using medical claims data is shown in Fig. 1. The first step involves matching diagnostic disease codes in the medical claims data to the corresponding disease names, which serve as predictors of activity limitations. The flow then branches into two arms. The left arm of the algorithm represents the process for estimating healthy life years and comprises: (i) generating the probability of individual activity limitation by using a machine-learning-based prediction model; (ii) calculating the prevalence of activity limitations for each age group; and (iii) estimating healthy life years at the municipal level by incorporating a life table. The right arm of the algorithm represents the process for estimating the prevalence of diseases that serve as predictors for healthy life years and comprises: (i) calculating the proportion of patients receiving care for each disease among the insured population; and (ii) estimating the prevalence of diseases affecting healthy life years without activity limitations at the municipal level. Details of each technique are described below.

### Disease-name matching

We extracted 5743 distinct diagnostic codes from outpatient medical claims, each classified according to the International Classification of Diseases, 10th Revision (ICD-10). In our previous research, we showed that activity limitations—an essential component in assessing healthy life years—can be reliably estimated based on a combination of demographic factors (age and sex) and the presence of 40 specific disease categories. These categories include, but are not limited to, mental health disorders such as depression, musculoskeletal conditions such as back pain and fractures, neurological issues including stroke, cerebral infarction, and Parkinson’s disease, as well as dementia, arthritis, and other types of injuries and chronic conditions [4]. The ICD-10 codes were matched and aggregated to the corresponding disease categories, which serve as predictors for activity limitation (Supplementary Table 1). All ICD-10 codes were cross-checked against the 40 disease categories used as predictors for activity limitation, and appropriate disease names were selected and matched. Codes deemed clinically inappropriate were excluded.

### Evaluation of healthy life years for each municipality

To estimate individual risks of activity limitations, we applied a machine-learning model based on the extreme gradient boosting algorithm, developed in a prior study [4]. The model incorporated predictors such as age, sex, and the presence of 40 disease categories under treatment. For each age group and sex, we computed the population-level prevalence of activity limitations by averaging individual probabilities and then applying a calibration process (Supplementary Figure 1). The correction coefficients were calculated by dividing the observed prevalence of activity limitations, derived from the CSLC, by the predicted rates from the claims-based model, stratified by age and sex. As the population structure of Kyoto Prefecture closely resembles that of Japan overall, this method is generalizable and could be used for the entire country (Supplementary Figure 2).

Municipal-level healthy life years without activity limitations were estimated by using Sullivan’s method, which incorporates the calibrated prevalence rates into life-table calculations [16, 17]. A life table was constructed as described in the Supplementary Methods. In brief, adjusted mortality rates and the average number of years lived within each age interval were estimated by using national life-table data and regional death and population statistics from Kyoto Prefecture. Subsequently, based on Chiang’s method, age-specific probabilities of death were derived from these estimates, and life-table indicators such as stationary population and life expectancy were sequentially calculated for each region.

### Evaluation of the prevalence of diseases affecting healthy life years

The proportions of patients receiving care for each disease were calculated among the insured population. The prevalence rate of diseases as predictors for healthy life years in each municipality was then calculated from the proportion of patients receiving care for each disease among the insured population following calibration. The correction coefficients were obtained by dividing the actual prevalence rate of disease for each sex, based on the CSLC, by the proportion of patients receiving care for each disease among the insured population.

## Results

**Figure 2:**
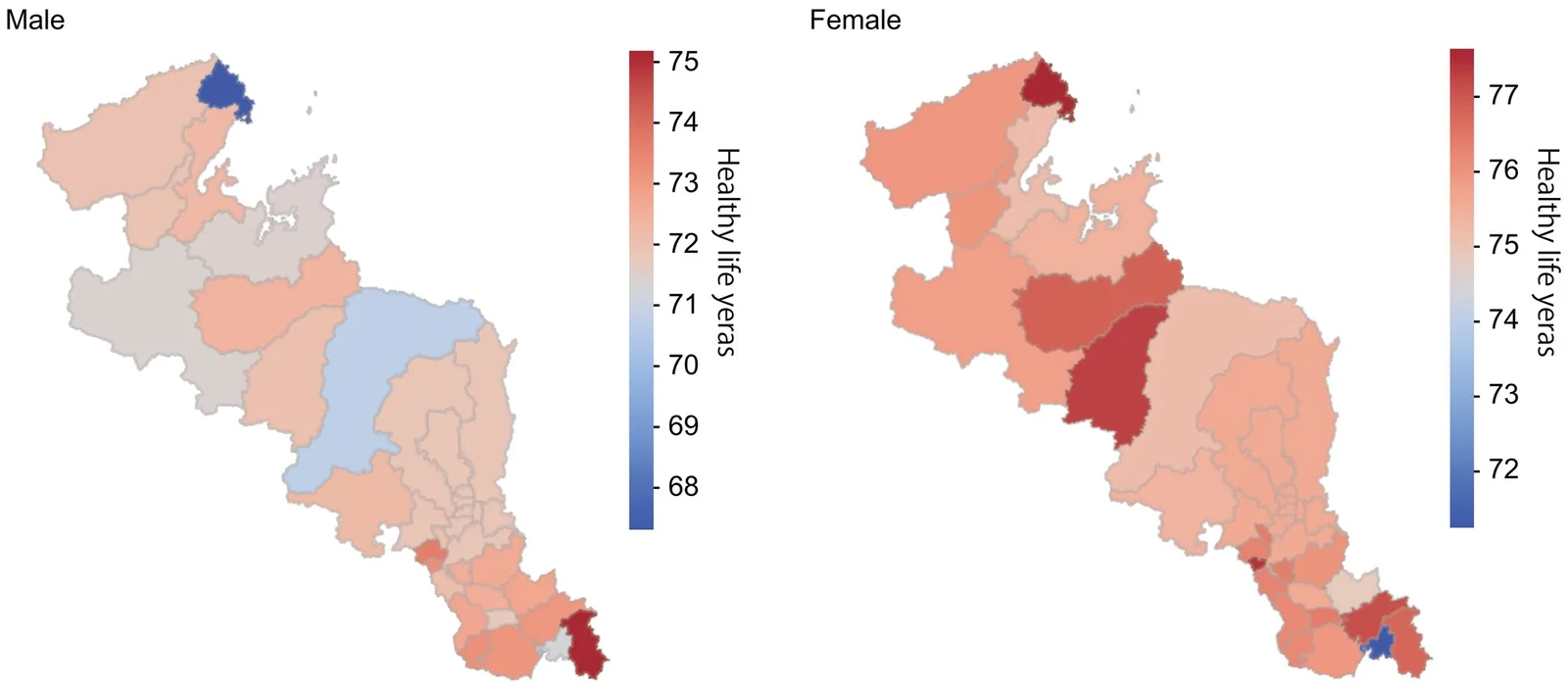
Healthy life years without activity limitations at the municipal level. The healthy life years of males and females in each municipality of Kyoto Prefecture as of June 2022 were estimated and visualized on a geographical map.

Municipal-level healthy life years without activity limitations, generated for both males and females as of June 2022, were visualized by using a geographic heat map (Fig. 2). Considerable variation was observed across municipalities: 72.1 years across all regions for males, ranging from 67.3 to 75.2 years, and 75.8 years for females, ranging from 71.3 to 77.6 years (Fig. 2 and Supplementary Table 2). The observed healthy life years for Kyoto Prefecture in 2022, as reported in the CSLC, were 72.1 years for males and 75.8 years for females, which were consistent with the estimated values.

**Figure 3:**
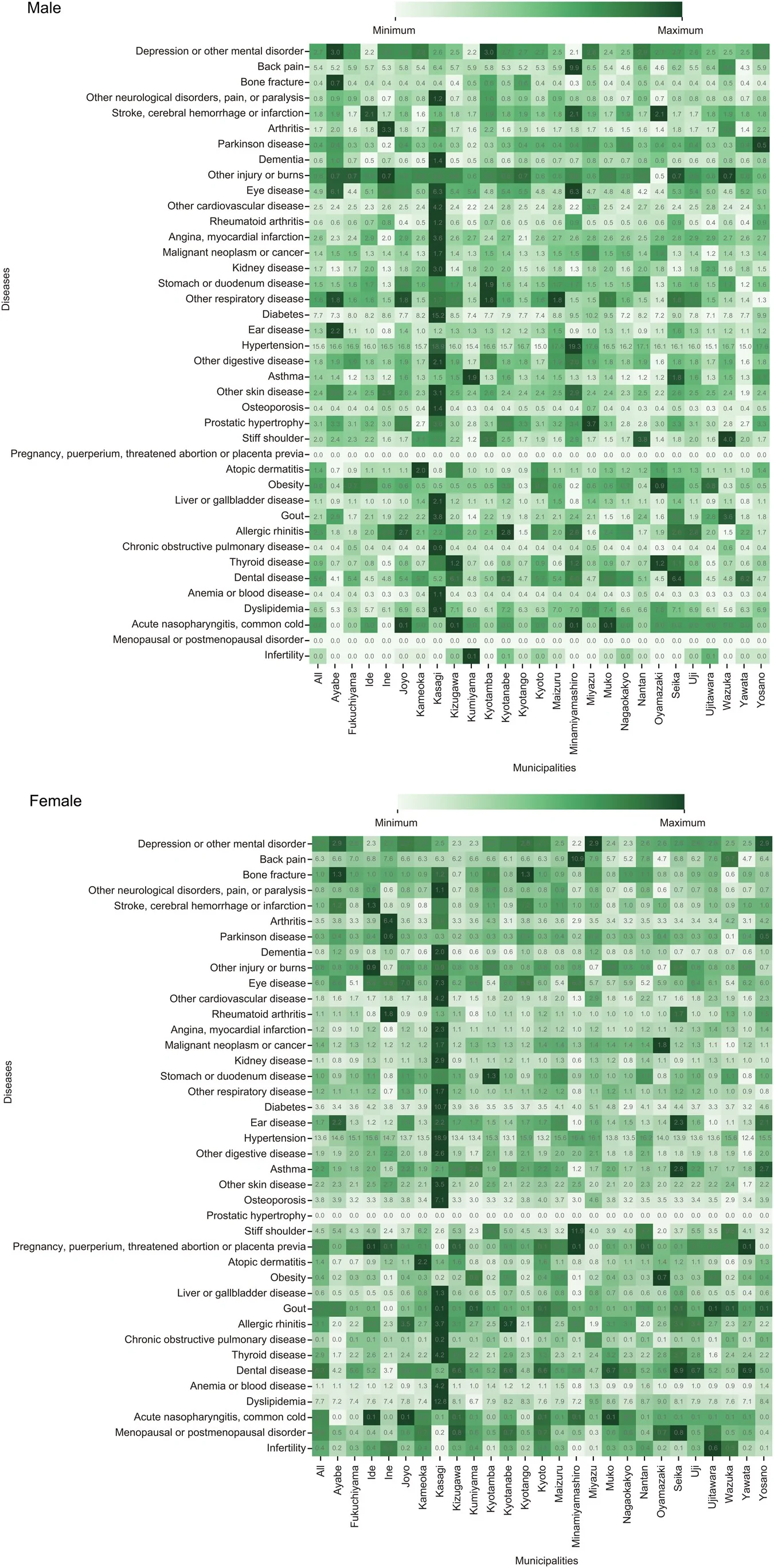
Prevalence of diseases affecting healthy life years without activity limitations at the municipal level. The prevalence of diseases affecting healthy life years in each municipality of Kyoto Prefecture as of June 2022 was estimated and visualized for males and females by using a heat map. The numbers in each cell indicate the prevalence rate of each disease. The diseases are ranked in descending order according to their impact on activity limitations.

The prevalence rates of diseases affecting healthy life years in each municipality of Kyoto Prefecture as of June 2022 were estimated and visualized for males and females by using a heat map (Fig. 3). The diseases were ordered by their impact on activity limitations according to our previous report [4]. The results also demonstrated regional disparities in disease prevalence. For example, the prevalence of depression or other mental disorders was 2.7% across all regions (ranging from 2.1% to 3.0%) for males and 2.7% (2.1%–3.0%) for females; back pain was 5.4% (4.3%–9.9%) for males and 6.3% (4.7%–10.9%) for females; bone fracture was 0.4% (0.3%–0.7%) for males and 1.0% (0.6%–1.3%) for females; and stroke was 1.8% (1.6%–2.1%) for males and 1.0% (0.8%–1.3%) for females (Fig. 3).

## Discussion

An estimation method for healthy life years without activity limitations was developed by using medical claims data at the municipal level. Diagnostic disease codes were matched and aggregated to the corresponding 40 disease categories, known as predictors for activity limitations. The probability of individual activity limitation was produced through a machine-learning-based prediction model. The prevalence rate of activity limitations in a population was then calculated. Healthy life years in each municipality as of June 2022 were estimated, along with the prevalence rates of diseases affecting healthy life years, showing the regional health disparities. This method enables real-time monthly evaluation of healthy life years at the municipal level, facilitating the rapid implementation of health-promotion measures tailored to regional characteristics.

Although the specific contributing conditions vary across municipalities, diseases with a high prevalence as estimated by using the present method, together with those having a high impact on activity limitations as demonstrated in our previous study [4], are considered to be the major contributors to the loss of healthy life years in each municipality.

When using a claims database, there are indeed issues related to diagnostic inaccuracy and population selection bias. However, in the method proposed in this study, we addressed these problems by performing calibration via data from the CSLC—a nationally representative survey conducted among a randomly selected general population. This approach was designed to mitigate these biases and more accurately reflect the characteristics of the general population.

Because healthy life years are estimated from the national CSLC survey conducted every 3 years, they cannot be evaluated in the intervening years between surveys. Additionally, there has been a waiting period of >2 years before the survey results are published. Healthy life years are typically calculated at the national, prefectural, and major-city levels, but no method existed for evaluating them at the municipal level. Our method makes it possible to calculate the real-time monthly healthy life years for each municipality and evaluate the prevalence rate of diseases affecting healthy life years.

In both Japan and many Western nations, estimates of healthy life years are generally derived from national surveys that assess activity limitations or self-perceived health conditions [12–14]. In contrast, the World Health Organization employs a different approach, calculating healthy life expectancy (HALE) based on subjective health assessments combined with disability weights for various health conditions, including diseases, injuries, and sequelae [3, 18, 19]. A study that was conducted in a Chinese city has explored the use of natural language processing to derive HALE estimates from electronic health records [20]. However, a method that objectively quantifies healthy life years specifically as the duration free from activity limitations—by using real-world medical data—has not been established. Our approach, which combines machine learning with routinely collected medical claims data, provides a novel and standardized way to estimate this metric without relying on infrequent or subjective surveys.

While our model was developed by using medical claims from Japan’s National Health Insurance system, its generalizability to other healthcare systems remains uncertain. Health insurance structures and population health profiles vary widely across countries, particularly in regions where the life expectancy is relatively short. Therefore, further studies are needed to assess the applicability and accuracy of the model in different international contexts.

## Conclusion

A novel estimation method for regional healthy life years without activity limitations was developed by using medical claims data. The real-time monthly evaluation of healthy life years at the municipal level, facilitating the rapid implementation of health-promotion measures tailored to regional characteristics, thereby contributes to the extension of healthy life years.

## Ethics approval

This study received ethical approval from the Kyoto Prefectural University of Medicine’s Institutional Review Board (number: ERB-C-2880). The research adhered to the ethical standards set forth in the Declaration of Helsinki. Because only anonymized health insurance and national survey data were used, the requirement for informed consent was waived.

## Supplementary Material

dyag126_Supplementary_Data

## Data Availability

Due to restrictions related to the nature of administrative data, public sharing of the dataset is not permitted. Data access may be requested through the Department of Health and Welfare, Kyoto Prefectural Government (https://www.pref.kyoto.jp/soshiki.html).
