# Supplementary material for: Estimating healthy life years without activity limitations using medical claims data in Japan
Source: Int J Epidemiol. 2026 Jul 30;55(4):dyag126. doi: 10.1093/ije/dyag126 (PMC13421775; doi:10.1093/ije/dyag126)

Supplementary Materials

"Estimating Healthy Life Years Without Activity Limitations Using Medical Claims Data in Japan"

Masahiro Nishi MD PhD[1], Reo Nagamitsu MD[2,3], Satomi Morita MD PhD[2,4], and Satoaki Matoba MD PhD[1]

[1]Department of Cardiovascular Medicine, Graduate School of Medical Science, Kyoto Prefectural University of Medicine, Kyoto, Japan

[2]Department of Health and Welfare, Kyoto Prefectural Government, Kyoto, Japan

[3]Department of Epidemiology for Community Health and Medicine, Graduate School of Medical Science, Kyoto Prefectural University of Medicine, Kyoto, Japan

[4]Department of Pulmonary Medicine, Graduate School of Medical Science, Kyoto Prefectural University of Medicine, Kyoto, Japan

Table of Contents

1. Supplementary Methods 2
   1.1 Life table construction 2
2. Supplementary Tables 4
   Supplementary Table S1. List of disease categories and corresponding ICD-10 codes. 4
   Supplementary Table S2. Estimated healthy life years at municipal level in Kyoto Prefecture as of June 2022. 5
3. Supplementary Figures 7
   Supplementary Figure S1. Elaboration for calculation method of prevalence rate of activity limitation for age group. 7
   Supplementary Figure S2. Population structure of Kyoto Prefecture and the entire country of Japan in 2022. 8

1. Supplementary Methods

1.1 Life table construction

Summary

First, adjusted mortality rates and average years lived within age intervals were estimated using national life table data and regional death and population statistics from Kyoto Prefecture. Subsequently, based on Chiang's method, age-specific probabilities of death were derived from these values, and life table indicators such as stationary population and life expectancy were sequentially calculated for each region.

Preparation for Life Table Construction

For a given region, let D_x and P_x represent the total number of deaths and the total population, respectively, for the age group x to x+n over the three-year period from year X-2 to year X. The mortality rate is then expressed as:

Mortality Rate = D_x/P_x

From the life table of year X, obtain the average number of years lived in the age interval x to x+n, denoted as a_x. It is assumed that a_x is universal across all regions. For the final age group (ω), set $a_\omega = 1$.

$$a_x = \frac{L_x - n \cdot l_{x+n}}{l_x - l_{x+n}}$$

Where:

- L_x: stationary population aged x to x+n

- l_x: number of survivors at age x

To ensure that the mathematical structure of the regional life table remains consistent with the national life table, a mortality rate adjustment factor, r_x, is introduced as a calibration factor for each age group. This factor represents the ratio of the observed national mortality rate to the theoretical rate derived from the national life table.

$$r_x = \frac{D_x^{nation}/P_x^{nation}}{(l_x - l_{x+n})/(T_x - T_{x+n})}$$

For the final age group, r_ω is calculated as:

$$r_\omega = \frac{D_\omega^{nation}/P_\omega^{nation}}{l_\omega/T_\omega}$$

Where:

- D_x^{nation}: national deaths for age x to x+n in year X

- P_x^{nation}: national population for age x to x+n in year X

- T_x: stationary population aged x and above

Calculate the adjusted mortality rate m_x for each age group in the region by dividing the regional mortality rate by the adjustment factor:

$$m_x = \frac{D_x/P_x}{r_x}$$

Life Table Calculation

Calculate the probability of death q_x for each age group using the age interval width n, the adjusted mortality rate m_x, and the average number of years lived a_x. For the final age group, set $q_\omega = 1$.

$$q_x = \frac{n \cdot m_x}{1 + n \cdot (1 - a_x/n) \cdot m_x}$$

Assuming the radix (initial number of survivors at age 0) is 100,000, calculate the number of deaths d_x in the age group x to x+n by multiplying the number of survivors l_x by the probability of death q_x. Survivors in the next age group are calculated recursively:

$$d_x = l_x \cdot q_x$$
$$l_{x+n} = l_x - d_x = l_x(1 - q_x)$$

Calculate the stationary population L_x for the age group x to x+n using a_x, n, l_x, and d_x. For the final age group, use $L_x = l_\omega/m_\omega$. The stationary population aged x and above, T_x, is the sum of L_x and subsequent age groups:

$$L_x = n \cdot l_x \cdot \{(1 - q_x) + a_x \cdot q_x\}$$
$$T_x = L_x + L_{x+n} + L_{x+2n} + \cdots + L_\omega$$

The life expectancy at age x, e_x, is given by:

$$e_x = T_x/l_x$$

The 95% confidence intervals (CIs) for life expectancy (LE) are calculated using the variance estimation method based on Chiang's variance formula and the normal approximation.

The variance of the probability of death for each age group, $V\{q_x\}$, is estimated as follows:

$$V\{q_x\} = \frac{q_x^2 \cdot (1 - q_x)}{D_x}$$

For the final age group, set $V\{q_\omega\} = 0$.

The variance of life expectancy at age x, $V\{e_x\}$, is estimated by:

$$V\{e_x\} = \frac{\sum_{y=x}^{\omega-n}\left[l_y^2 \cdot \left\{\left(1-a_y\right)n_y + e_{y+n_y}\right\}^2 \cdot V\{q_y\}\right]}{l_x^2}$$

For the final age group, the variance is calculated as:

$$V\{e_\omega\} = \frac{1-m_\omega}{D_\omega \cdot m_\omega^2}$$

The 95% CI for life expectancy is then given by:

$$e_x \pm 1.96 \cdot \sqrt{V\{e_x\}}$$

2. Supplementary Tables

Supplementary Table S1. List of disease categories and corresponding ICD-10 codes.
All ICD-10 codes were cross-checked against the 40 disease categories used as predictors for activity limitation, and appropriate disease names were selected and matched. Codes deemed clinically inappropriate were excluded.

An example subset of list is provided below. The complete list is included in the attached file "Supplementary_Table_S1_Disease_Categories_and_ICD10_Codes.xlsx."

No	Disease or injury	ICD10 code (three digits)	ICD10 code (full)
1	Diabetes	E10	E100
			E101
			E102
			E103
			E104
			E105
			E106
			E107
			E108
			E109
		E11	E110
			E111
			E112
			E113
			E114
			E115
			E116
			E117
			E118
			E119
		E12	E120
			E121
			E122
			E123
			E124

			E125
			E126
			E127
			E128
			E129
		E13	E130
			E131
			E132
			E133
			E134
			E135
			E136
			E137
			E138
			E139
		E14	E140
			E141
			E142
			E143
			E144
			E145
			E146
			E147
			E148
			E149
2	Obesity	E66	E660<br>E661<br>E662<br>E668<br>E669

Supplementary Table S2. Estimated healthy life years at municipal level in Kyoto Prefecture as of June 2022.

Municipality	Male		Female	
	HLY	95% CI	HLY	95% CI
All	72.14	72.01－72.26	75.78	75.67－75.9
Yosano	71.94	70.54－73.34	75.95	74.56－77.33
Kumiyama	72.52	71.08－73.95	76.4	75.13－77.67
Kameoka	72.22	71.52－72.91	75.38	74.69－76.06
Ide	71.77	69.89－73.64	76.44	74.66－78.22
Kyotango	72.01	71.12－72.89	75.94	75.11－76.77
Kyotamba	72.04	70.48－73.6	77.27	75.94－78.6
Kyotanabe	72.7	71.91－73.48	76.21	75.48－76.94
Kyoto	71.88	71.72－72.05	75.58	75.43－75.74
Ine	67.31	58.85－75.77	77.64	73.3－81.99
Yawata	72.15	71.42－72.88	76.31	75.64－76.98

Nantan	70.78	69.55－72.01	75.19	74.14－76.25
Minamiyamashiro	75.19	72.26－78.11	76.8	71.47－82.12
Muko	72.05	71.16－72.94	76.43	75.65－77.21
Wazuka	73.07	70.68－75.47	77.1	74.94－79.27
Joyo	72.52	71.79－73.24	75.59	74.86－76.31
Oyamazaki	73.26	71.49－75.04	77.43	75.98－78.88
Uji	72.62	72.17－73.07	76	75.55－76.45
Ujitawara	72.76	70.89－74.64	74.84	72.75－76.93
Miyazu	72.24	70.84－73.65	75.19	73.53－76.85
Kizugawa	73.1	72.39－73.81	75.94	75.26－76.61
Fukuchiyama	71.42	70.62－72.22	75.81	75.06－76.57
Kasagi	71.23	64.96－77.51	71.25	64.83－77.67
Seika	73.24	72.09－74.39	76.22	75.26－77.19
Ayabe	72.37	71.22－73.52	76.86	75.8－77.93
Maizuru	71.46	70.69－72.23	75.41	74.68－76.14
Nagaokakyo	73.65	72.91－74.39	76.33	75.63－77.03

HLY: healthy life years, CI: confidence interval.

3. Supplementary Figures

Supplementary Figure S1. Elaboration for calculation method of prevalence rate of activity limitation for age group.

Prevalence rate of activity limitation in a population group was calculated following the averaging the individual probability of activity limitations and calibration using correction coefficients. A graph shows prevalence rates of activity limitations for age group of each sex in a municipality

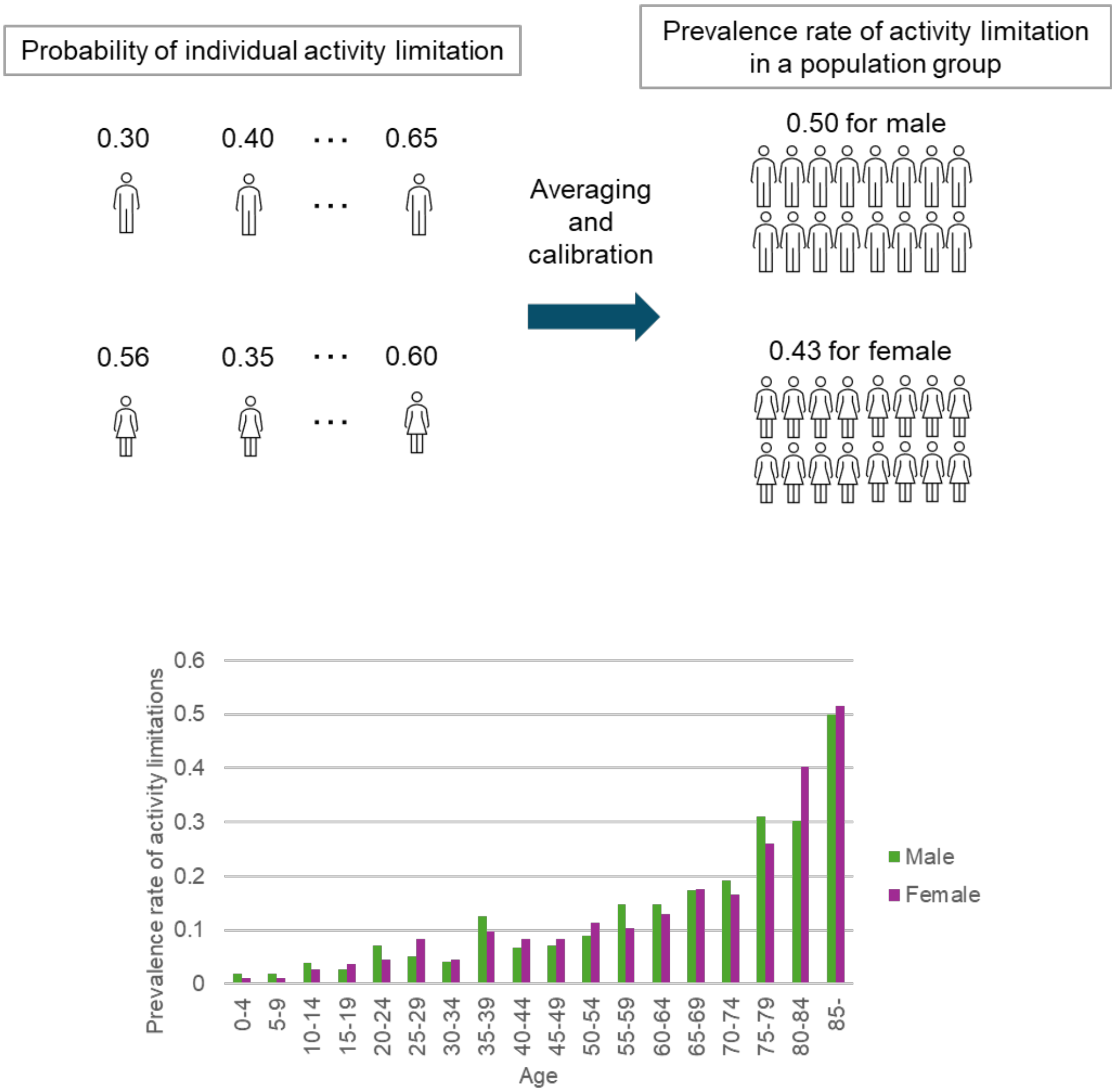

Supplementary Figure S2. Population structure of Kyoto Prefecture and the entire country of Japan in 2022.

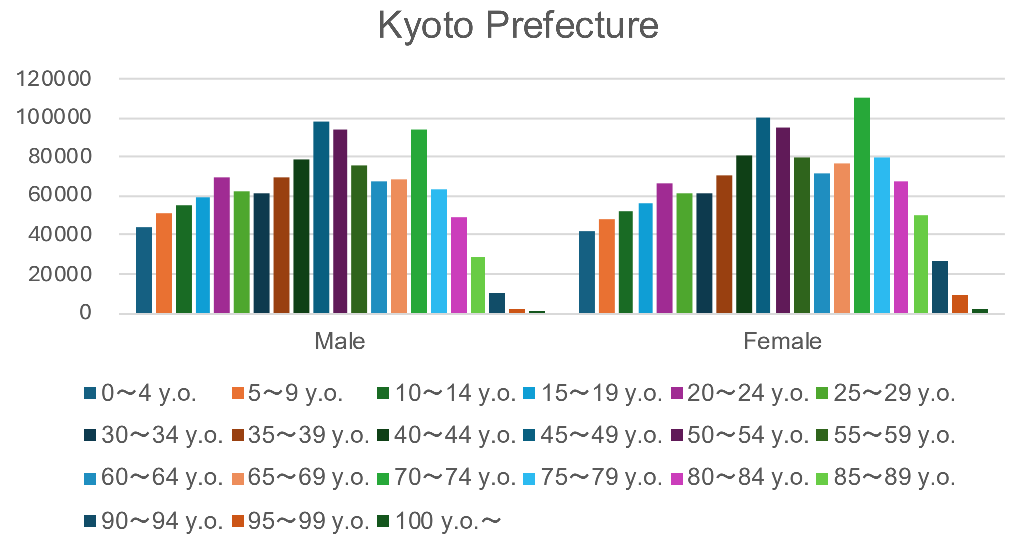

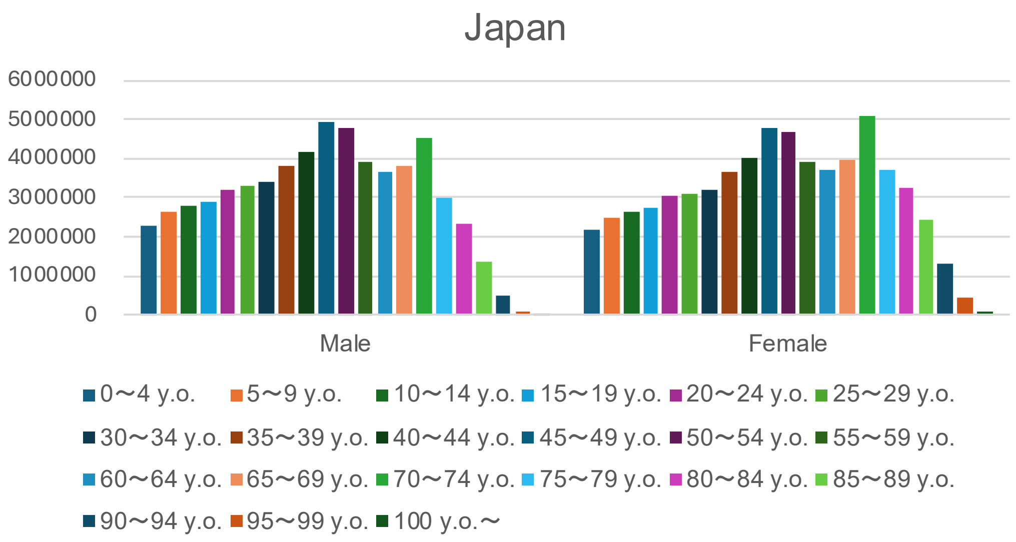

Population pyramid: Japan vs Kyoto Prefecture

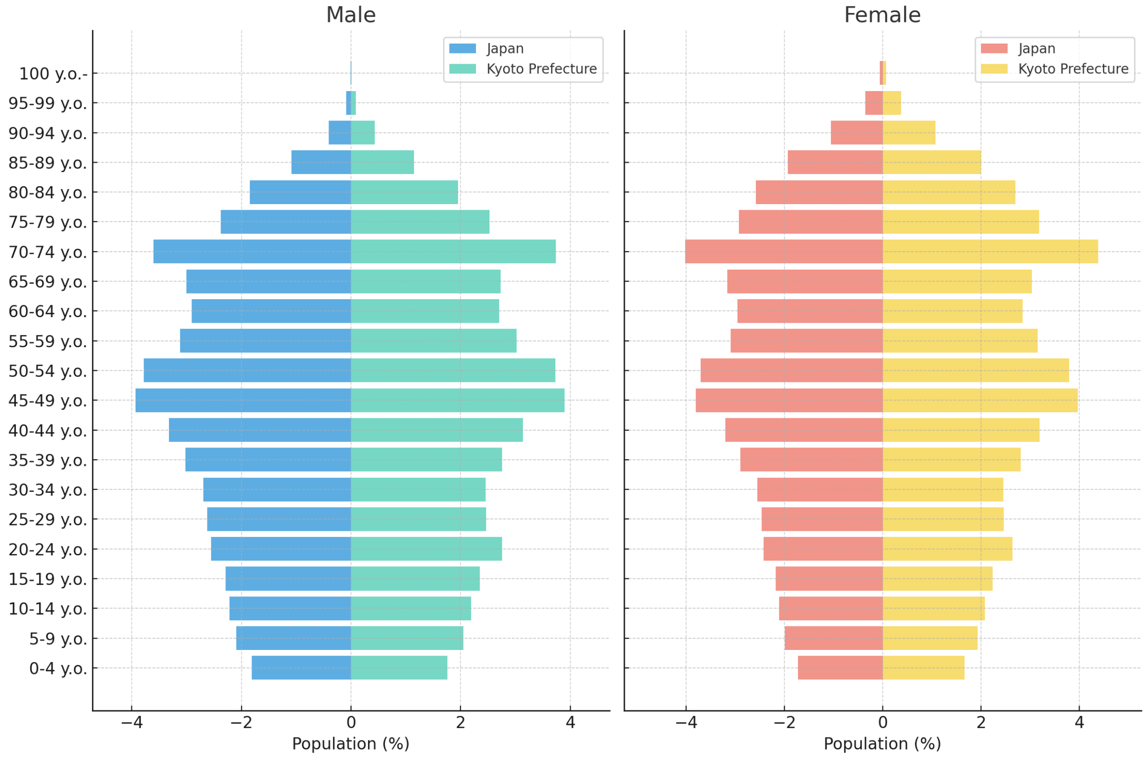

Supplement: dyag126_Supplementary_Data [file dyag126_supplementary_data.zip › ije-2025-07-1297-File006.pdf]
